# Role of Constitutive Behavior and Tumor-Host Mechanical Interactions in the State of Stress and Growth of Solid Tumors

**DOI:** 10.1371/journal.pone.0104717

**Published:** 2014-08-11

**Authors:** Chrysovalantis Voutouri, Fotios Mpekris, Panagiotis Papageorgis, Andreani D. Odysseos, Triantafyllos Stylianopoulos

**Affiliations:** 1 Cancer Biophysics laboratory, Department of Mechanical and Manufacturing Engineering, University of Cyprus, Nicosia, Cyprus; 2 EPOS-Iasis R&D, Division of Biomedical Research, Nicosia, Cyprus; 3 Biomedical Imaging Laboratory, Department of Electrical and Computer Engineering, University of Cyprus, Nicosia, Cyprus; University of Nebraska Medical Center, United States of America

## Abstract

Mechanical forces play a crucial role in tumor patho-physiology. Compression of cancer cells inhibits their proliferation rate, induces apoptosis and enhances their invasive and metastatic potential. Additionally, compression of intratumor blood vessels reduces the supply of oxygen, nutrients and drugs, affecting tumor progression and treatment. Despite the great importance of the mechanical microenvironment to the pathology of cancer, there are limited studies for the constitutive modeling and the mechanical properties of tumors and on how these parameters affect tumor growth. Also, the contribution of the host tissue to the growth and state of stress of the tumor remains unclear. To this end, we performed unconfined compression experiments in two tumor types and found that the experimental stress-strain response is better fitted to an exponential constitutive equation compared to the widely used neo-Hookean and Blatz-Ko models. Subsequently, we incorporated the constitutive equations along with the corresponding values of the mechanical properties - calculated by the fit - to a biomechanical model of tumor growth. Interestingly, we found that the evolution of stress and the growth rate of the tumor are independent from the selection of the constitutive equation, but depend strongly on the mechanical interactions with the surrounding host tissue. Particularly, model predictions - in agreement with experimental studies - suggest that the stiffness of solid tumors should exceed a critical value compared with that of the surrounding tissue in order to be able to displace the tissue and grow in size. With the use of the model, we estimated this critical value to be on the order of 1.5. Our results suggest that the direct effect of solid stress on tumor growth involves not only the inhibitory effect of stress on cancer cell proliferation and the induction of apoptosis, but also the resistance of the surrounding tissue to tumor expansion.

## Introduction

Mechanical solid stresses (i.e, the stresses of the solid phase of a tumor) shape the tumor microenvironment by at least two ways: directly by compressing cancer and stromal cells and indirectly by compressing intratumor blood and lymphatic vessels [1]–[4]. Compression of cancer cells reduces their proliferation rate, induces apoptosis and enhances their invasive and metastatic potential [5]–[9]. Therefore, tumors that exhibit higher stress levels might have lower growth rates and a higher propensity to metastasize [2], [5], [7], [10]–[13]. Compression of stromal cells can convert fibroblasts into myo-fibroblasts, known as cancer-associated fibroblasts, that produce extracellular proteins (e.g., collagen, fibronectin, tenascin-C) and cause a desmoplastic reaction [14]–[17]. Additionally, compression of blood vessels reduces tumor perfusion and supply of oxygen and nutrients, which often causes the formation of necrotic tissue at the center of the tumor. Also, it can drastically decrease the delivery of blood-borne therapeutic agents and thus, compromise the efficacy of chemo- and nano-therapies [2], [18], [19]. Finally, compression of lymphatic vessels contributes to the uniform elevation of the interstitial fluid pressure due to the inability of the tumor to drain the excessive interstitial fluid [17]. Interstitial hypertension in turn, is a major barrier to the delivery of drugs [20].

Despite the importance of solid stresses on tumor progression and treatment, there is no technique to date to measure solid stress levels *in vivo*. Therefore, calculations of the evolution and magnitude of stress in tumors is performed only with the use of mathematical modeling. Biomechanical models of tumor growth have been developed by our research group and other researchers, which have been successful in predicting qualitatively the overall mechanical response of tumors [2], [3], [21]–[25]. These models, however, face certain limitations that might affect the accuracy of the calculations. There are very few studies to measure the mechanical properties (e.g. stiffness) of solid tumors [26]–[28] and little work has been performed for their constitutive modeling compared to other biological tissues such as arteries and heart valve leaflets. As a result, general constitutive equations, particularly the neo-Hookean and the Blatz-Ko material, are being used to describe the elastic response of tumors with limited experimental validation and with the mechanical properties taken either arbitrarily or from the few published experimental data. Therefore, the uncertainty of the selection of a proper constitutive equation as well as the corresponding mechanical properties can compromise the accuracy of biomechanical models and thus, our ability to better understand the tumor mechanical microenvironment. Furthermore, while it has been shown with *in vitro* experiments in cancer cell spheroids that the mechanical properties of the host tissue affect the state of stress and the growth of the tumor [5], [7], the underlying mechanisms for the mechanical interactions between the tumor and the host tissue are not clear yet.

To this end, we performed unconfined compression experiments of tumor specimens in a dynamic mechanical analysis system to characterize their response. We tested two different tumor types and measured the elastic modulus from the experimental stress-strain curves and tan(*δ*), the ratio of the elastic to the loss modulus. Subsequently, in order to determine a proper constitutive equation and the corresponding values of the mechanical properties, we simulated the experimental procedure and fitted the stress-strain data to three constitutive models: the neo-Hookean and the Blatz-Ko models that are widely used for tumors and an exponential equation that has been previously developed to model soft biological tissues. Finally, we incorporated this information into a biomechanical model of tumor growth to study the evolution of solid stress in tumors during progression and the contribution of the host tissue to the tumor’s state of stress and growth rate.

## Materials and Methods

### Cell culture and in vivo tumor models

Two cancer cell lines were used in this study: the human breast cancer cell line MCF10CA1a, derived from *in vivo* passaging of H-Ras transformed MCF10A cells [29] and the metastatic human colon adenocarcinoma cell line SW620 (ATCC). Cells were maintained in culture under standard conditions (37°C, 5% CO_2_ and 95% humidity) until 75–80% confluent. Cultures were then trypsinized, washed twice and resuspended in HANK’s balanced media. Tumors were prepared by implanting 3×10^6^ SW620 cells or 5×10^5^ MCF10CA1a cells suspended in 100 µL HANK’s balanced media into immunodeficient CD1 nude female mice. The mice (two groups of 7–8 week-old, 8 animals/group) were housed and cared in a pathogen–free environment in accordance to the European Commission Recommendations 2007/526 and the European Directive 2010/63. Cell suspensions were injected subcutaneously in the right flank of each animal.

Tumor growth was monitored on a daily basis and its planar dimensions (*x*, *y*) were measured with a digital caliper every 2 days. Tumor volume was measured from its planar dimensions using the volume of an ellipsoid and assuming that the third dimension, *z*, equals to 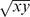 [2]. Therefore, the volume was given by the equation: 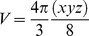, which yields 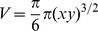. When tumors reached approximately 10 mm (520 mm^3^) in size, mice were sacrificed with cervical dislocation and tumors were excised and placed in culture medium. Other humane endpoints were when animals would exhibit signs of distress or excessive loss of weight (>10%), but no such a case was observed in our experiments. In general, 10 to 20 days elapsed between when the mice were first injected with the SW620 or MCF10CA1a cells and when they were sacrificed. Six animals (*n* = 6) gave tumors in the SW620 group and seven animals (n = 7) gave tumors in the MCF10A1a group. All specimens were tested within an hour from excision. The experiments were conducted in strict accordance with the animal welfare regulations and guidelines of the Republic of Cyprus and the European Union under a license acquired by the Cyprus Veterinary Services (No CY/EXP/PR.L6/2012), the Cyprus national authority for monitoring animal research.

### Histological analysis

Tumors were isolated from the CD1 nude mice injected either with MCF10CA1a or SW620 cancer cells, fixed in 4% paraformaldehyde and embedded in paraffin. Tumor tissue sections (10 µm-thick) were performed with a Leica RM2125RT microtome, followed by staining with Hematoxylin and Eosin (H&E) using standard methodology. Stained slides were observed using Axiovert 200 M inverted microscope (Carl Zeiss) and bright-field images were acquired at 20X magnification with the AxioVision 4.4 software (Figure S1 in File S1).

### Unconfined compression experiment

The unconfined compression test was carried out using a commercial dynamic mechanical testing system (Triton Technology, Lincolnshire, UK). The specimens were cut in an orthogonal shape with dimensions 8×8×6 mm (length×width×thickness). We followed a strain scan experimental protocol according to which the tumor was compressed stepwise to a maximum strain of 12% and the developed force was recorded. At each strain step the system performed a dynamic analysis to measure tan(*δ*) from the ratio of the elastic to the loss modulus. To ensure that the force values we obtained did not include significant viscous effects, we used a low frequency of 1 Hz, according to the instructions of the manufacturer. Furthermore, tan(*δ*) was found to be on the order of 0.1 for both tumor types, which is relatively low and reveals a more solid-like than viscous behavior. Subsequently, we converted the force data to the 1^st^ Piola-Kirchhoff stress and the displacement data to infinitesimal strain (i.e., 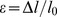) and calculated the elastic modulus of the tumors from the linear part of the stress-strain curves. The values of the elastic modulus obtained with this method were similar (less than 5% deviation) to the elastic modulus measured by the system during the dynamic testing, which verified that the force measurements did not include significant viscous effects.

### Statistical analysis

The data are presented as means with standard errors. Experimental groups were compared using an unpaired Student’s t-test. Statistical significant difference was determined when p<0.05.

### Biomechanical modeling of tumor growth

**Figure 1:**
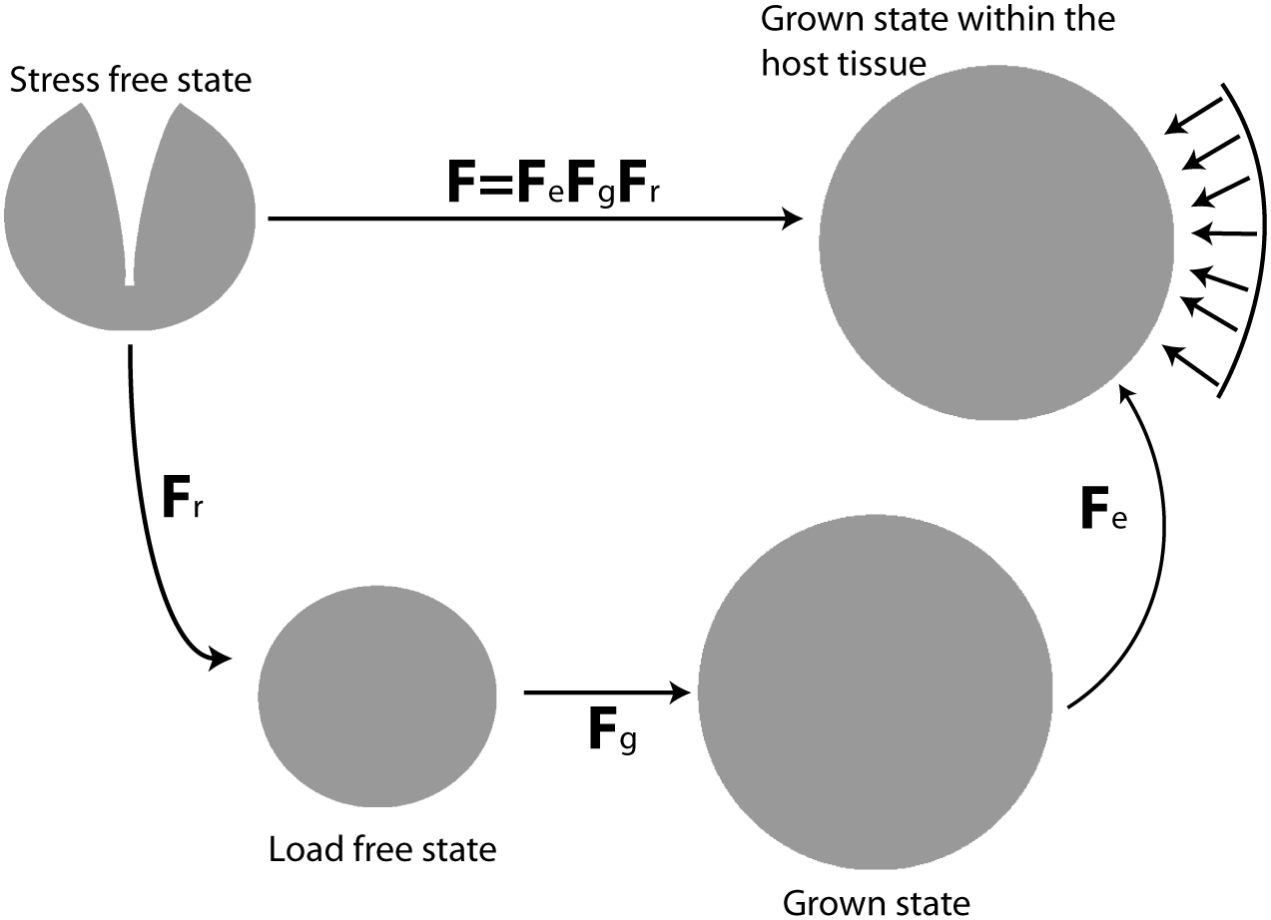
Multiplicative decomposition of the deformation gradient tensor. The stress free state corresponds to an excised tumor having the growth-induced (residual) component of the solid stress released [2], [3]. The load free state corresponds to an excised tumor currying no external loads but holds residual stress, described by **F** *_r_*. The grown state corresponds to the volumetric growth of the tumor, which is described by **F** *_g_* and the grown state within the host tissue corresponds to the final configuration of the grown tumor accounting for external stresses (arrows) by the host tissue and described by **F** *_e_*.

Solid stress in a tumor has two components: the residual “growth-induced” stress, which accumulates in tumors due to the generation of internal forces among the structural constituents of the tumor and the externally applied stress due to mechanical interactions with the host tissue [3]. To model the growth and mechanical behavior of tumors the multiplicative decomposition of the deformation gradient tensor, **F**, was used [30], a methodology that has been applied successfully to solid tumors [3], [21], [25], [31]–[33] as well as to other soft tissues [34], [35]. The model considered only the solid phase of the tumor and accounted for i) tumor growth, ii) generation of residual, growth-induced stresses and iii) mechanical interactions between the tumor and the surrounding normal tissue. Therefore, **F** was divided into three components:
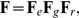
(1)where **F** *_e_* is the elastic component of the deformation gradient tensor and accounts for mechanical interactions with the surrounding normal tissue or with any other external stimulus, **F** *_g_* is the component that accounts for tumor growth and **F** *_r_* is the component that accounts for the generation of residual stresses (Figure 1).

We measured and quantified residual stresses in tumors in previous research [2], [3]. In this analysis, tumors did not exhibit significant levels of residual stress, and thus we set 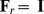.

Tumor growth was taken to be isotropic and the growth component **F** *_g_* was given by:
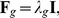
(2)where *λ_g_* is the growth stretch ratio. The growth stretch ratio was described by a phenomenological Gompertzian equation expressed in differential form as [3]:
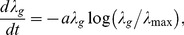
(3)where *t* is the time, *a* describes the growth rate of the cells and *λ_max_* is a maximum growth stretch ratio. We further assumed that the parameter *a* depends on the levels of solid stress through a linear relationship [22]:



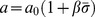
(4)where *a*_0_ is the growth rate at zero stress, *β* is a constant that describes the dependence of *a* on stress and 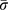 is the bulk solid stress calculated as the average of the radial, 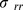, and circumferential (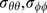) components of the Cauchy stress tensor (i.e., 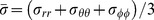).

Finally, three constitutive equations, commonly used for the elastic response of soft biological tissues were tested: the compressible neo-Hookean model, the Blatz-Ko model and an exponential constitutive equation [36]. The strain energy density functions, *W*, of the equations are:

neo-Hookean: 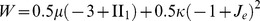
(5)


Blatz-Ko: 
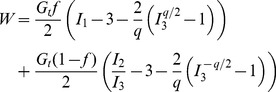
(6)


exponential: 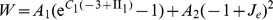
(7)where the mechanical properties of the neo-Hookean model are the shear modulus *µ* and bulk modulus *κ*. The properties of the Blatz-Ko model are the shear modulus *G_t_*, 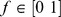 and *q*<0 are dimensionless parameters. For isotropic materials 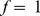, and thus only the first term of the right hand side of Eq. 6 was considered in our study. The parameters of the exponential equation are the constants *A*_1_, *A*_2_ and *C*_1_. *J_e_* is the determinant of the elastic deformation gradient tensor **F** *_e_* and 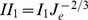. *I_1_*_,_ *I_2_* and *I_3_* are the invariants of the right Cauchy-Green deformation tensor, which is evaluated from the elastic part of the deformation gradient tensor, **F**_e_.

The Cauchy stress tensor, **σ**, was calculated by the strain energy density function as [37]:
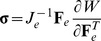
(8)


Finally, the linear momentum balance was solved assuming a quasi-static problem in the absence of body forces:
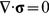
(9)


A finite element model was constructed in COMSOL to solve Eqs. 1–9. A detailed description of the implementation of the model can be found in File S1.

## Results

### *Ex vivo* stress-strain response is highly nonlinear

**Figure 2:**
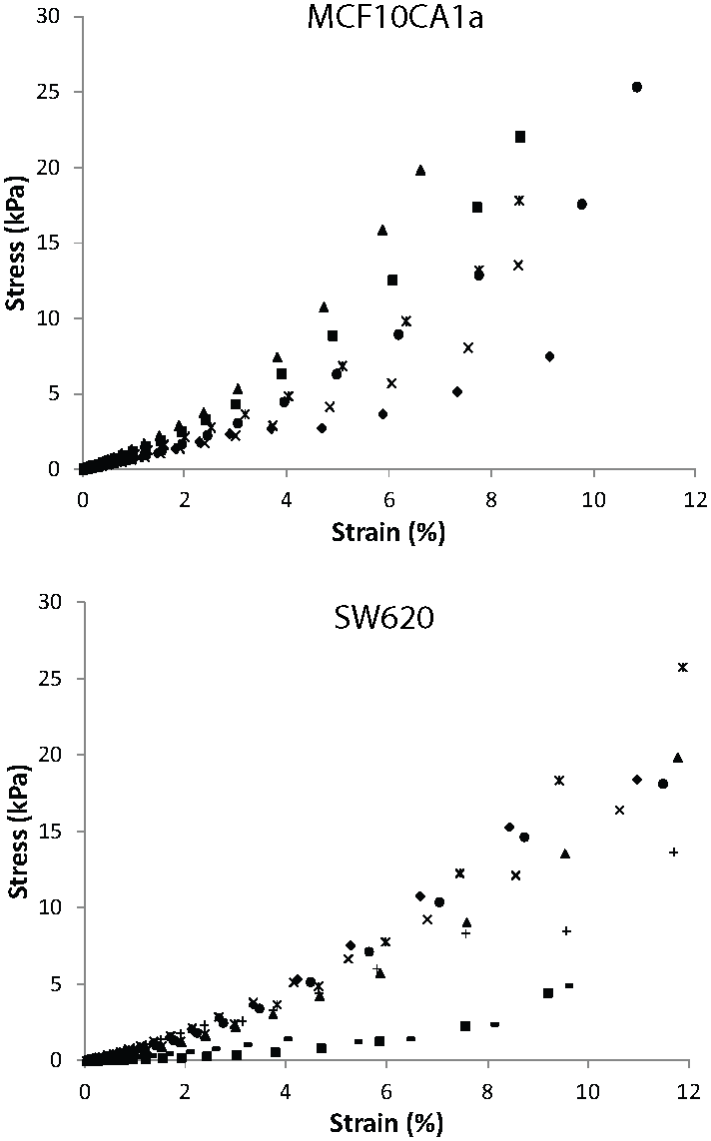
Stress-strain response of tumors. Experimentally measured elastic stress-strain response of MCF10CA1a and SW620 tumors in unconfined compression. Data show individual tumor behavior.

The experimental results of the unconfined compression experiments for the individual tumors and for the two tumor types are shown in Figure 2. The equilibrium stress-strain response is highly nonlinear in most of the specimens even at the low range of strain employed in the study. The data are typical of soft biological tissues, consisting of a toe region at lower strains and a linear region at higher strains. The elastic modulus was measured from the slope of the linear part of the curves and found to be 288.3±45.5 kPa for the MCF10CA1a tumors and 186.1±25.9 kPa for the SW620 tumors. The difference between the two groups was not statistically significant (p = 0.065). The values of tan(*δ*) was 0.108±0.009 and 0.098±0.011 for the MCF10CA1a and SW620, respectively (Table 1). The results were not statistically different (p = 0.106).

**Table 1:** Experimental measurements for the elastic modulus and tan(*δ*) of the two tumor types.

Tumor Type	Elastic Modulus (kPa)	tan(*δ*)
MCF10CA1a	288.3±45.5	0.108±0.009
SW620	186.1±25.9	0.098±0.011

### An exponential constitutive equation better describes tumor’s *ex vivo* mechanical behavior

**Figure 3:**
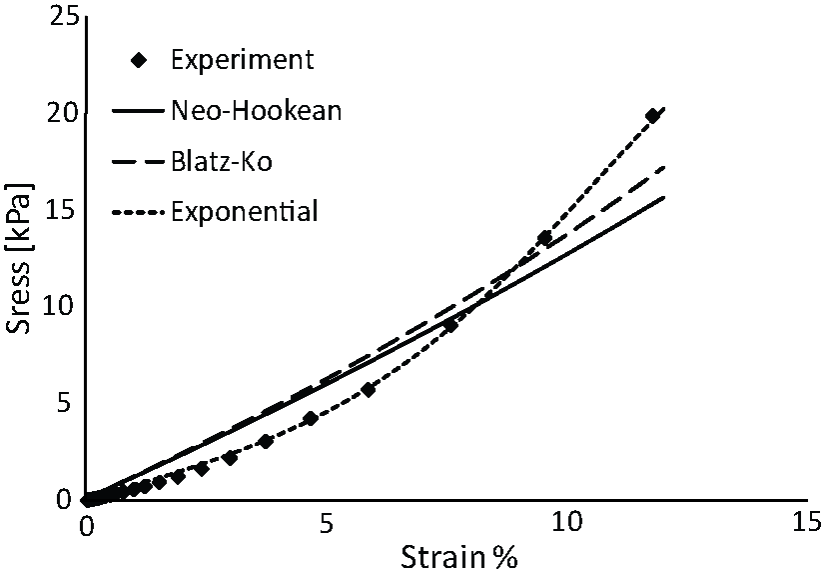
Fitting to experimental data. Representative fitting of the neo-Hookean, Blatz-Ko and exponential constitutive equations to the experimentally measured stress-strain response of a tumor specimen.

Subsequently, the biomechanical model was employed to simulate the unconfined compression experiment in order to indentify the most suitable constitutive equation as well as the values of the mechanical properties that provide the best fit to the experimental data. For these simulations 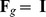, because the experiment was performed *ex vivo* and thus, 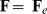 in Eq. 1 describes the elastic deformation of the tumor during the simulated compression experiment. A three-dimensional finite element model of orthogonal geometry, same in size as that of the real specimens, was constructed consisting of 10,563 tetrahedral finite elements. The model was compressed in one direction and was free to deform in the other two according to the unconfined compression experimental protocol. The mechanical properties of the constitutive equations were varied so that the sum of the squared errors, 
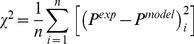
, reached a minimum. 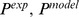 are the experimentally measured and predicted by the model 1^st^ Piola-Kirchhoff stresses, respectively, and *n* the number of experimental data. Figure 3 shows a typical fit of the model. The exponential constitutive equation describes the response of the tissue very well, while the neo-Hookean and Blatz-Ko models cannot capture the highly nonlinear response of the tumor. The average values and standard errors of the model parameters and the values of *χ* ^2^ are shown in Table 2.

**Table 2:** Values of the mechanical properties of the two tumor types derived by fitting the model to the experimental stress-strain curves.

Tumor type	neo-Hookean (Eq. 5)^a^			Blatz-Ko (Eq. 6)			Exponential (Eq. 7)			
	*µ* (kPa)	*Κ* (kPa)	*χ* ^2^	*G_t_* (kPa)	*q*	*χ* ^2^	*A*_1_ (Pa)	*A*_2_ (kPa)	*C*_1_	*χ* ^2^
MCF10CA1a	37.8 (6.5)	50.4 (8.6)	0.82 (0.13)	28.9 (6.2)	1.52 (0.46)	0.66 (0.11)	115.2 (23.7)	31.7 (7.9)	43.3 (3.0)	0.19 (0.04)
SW620	26.7 (6.1)	35.5 (8.1)	1.41 (0.20)	22.1 (6.6)	2.44 (1.01)	0.71 (0.14)	100.2 (25.1)	18.7 (2.1)	40.3 (5.1)	0.30 (0.04)

Standard errors are shown in parenthesis.

a The Poisson’s ratio was taken to be 0.2 [22].

### The selection of tumor’s constitutive equation has a minor effect on the *in vivo* evolution of solid stress during progression

To model tumor growth, the tumor was represented as a sphere with an initial diameter of 500 µm surrounded by normal tissue. Tumors of this size are large enough to be treated as a continuum and at this size the stresses start to evolve [38]. The normal tissue had a cubic shape and its size was two orders of magnitude larger than that of the tumor to avoid boundary effects. The surrounding host tissue was modeled as a neo-Hookean material with a shear modulus of *µ* = 15 kPa and a Poisson’s ratio of *ν* = 0.2 [3], [36]. Due to symmetry, we solved for the one eighth of the domain, applying a symmetry boundary condition at the symmetric boundaries and a stress free condition at the free surfaces (Figure S2 in File S1). The finite element software accounts automatically for the continuity of the surface tractions and the displacements at the interface of the tumor with the normal tissue. The domain consisted of 49,668 tetrahedral finite elements.

**Figure 4:**
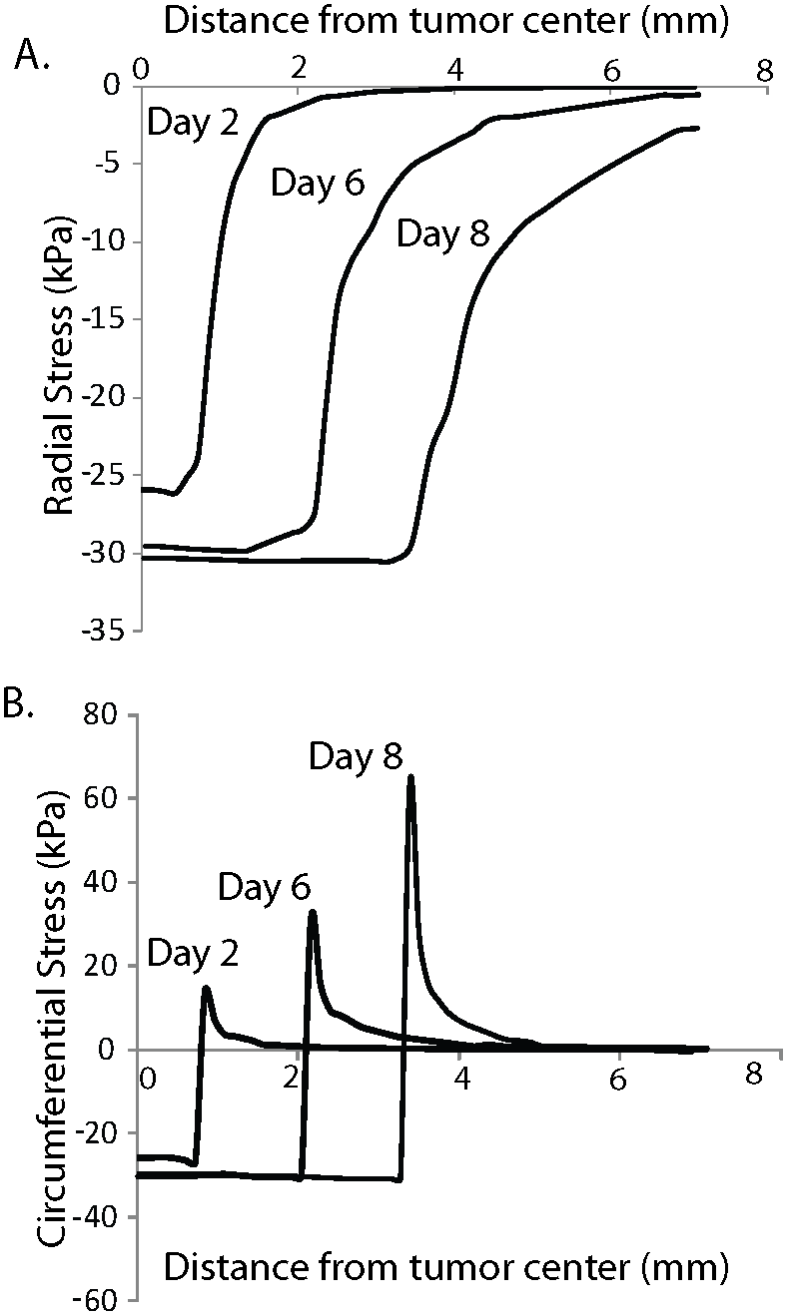
Spatial profile of solid stress. The A) radial and B) circumferential solid stress in the tumor and the surrounding normal tissue are presented at three different time points. Solid stress is compressive and uniform in the tumor interior, while at the interface with the normal tissue radial stress diminishes and circumferential stress turns steeply to tensile.

**Figure 5:**
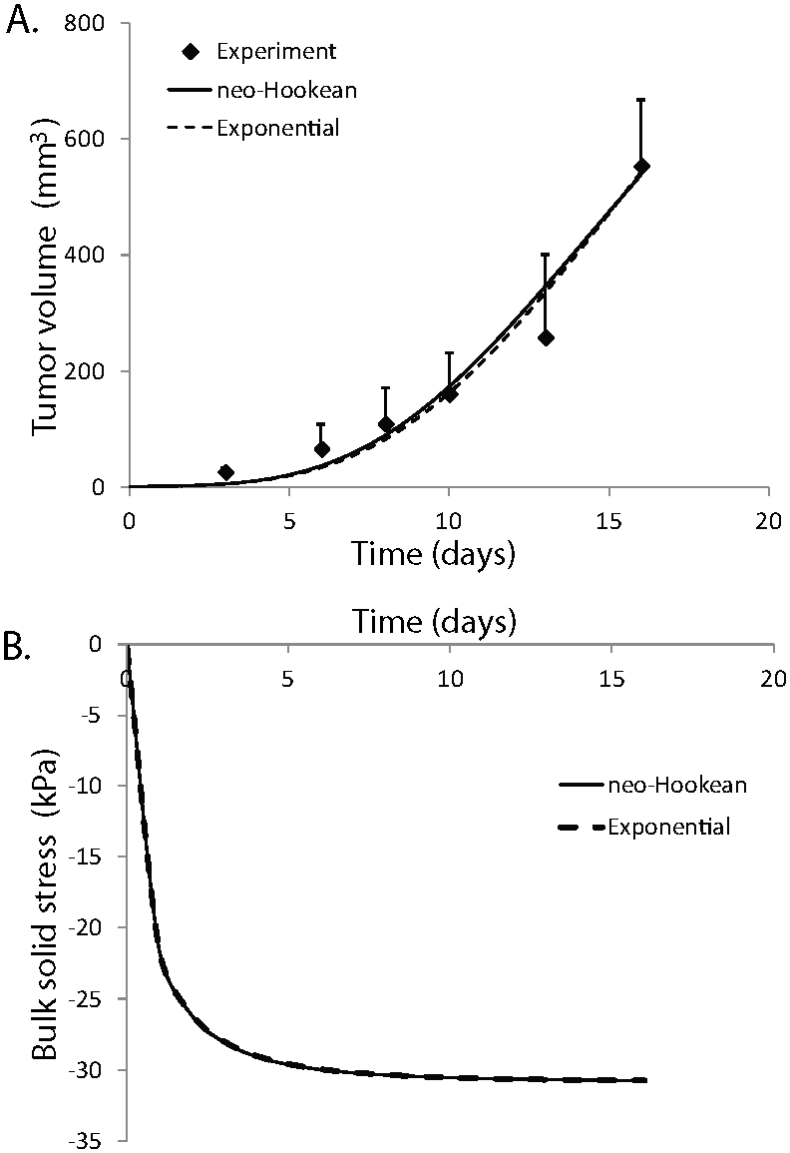
Effect of tumor constitutive behavior on tumor growth and state of stress. A) Model fit to the experimentally measured growth curve of SW620 tumors using the neo-Hookean and the exponential equation. B) Evolution of bulk solid stress in the tumor interior does not depend on the selection of the constitutive equation. Results using the Blatz-Ko material are omitted for clarity.

The assumption of isotropic tumor growth results in a uniform evolution of compressive solid stress within the tumor, while at the interface with the normal tissue the radial stress diminishes while the circumferential stress turns to tensile (Figure 4). These predictions for the spatial distribution of the solid stress are in agreement with previous mathematical models [22], [23]. To specify the parameters *λ_max_*, *α* and *β* (Eqs. 3 and 4), we fitted the model to the experimental growth data of the SW620 tumors. Interestingly, the values of these parameters were independent from the choice of the constitutive equation when the mechanical properties presented in Table 2 were used (Figure 5A). The values were *λ_max_* = 50, *α* = 0.42 day^−1^ and *β* = 2.2×10^−5^ Pa^−1^ for all three constitutive equations. Moreover, the stresses developed in the tumor did not depend on the selection of the constitutive equation (Figure 5B). Therefore, despite the fact that in the *ex vivo* situation the three constitutive equations provide largely different predictions, with the exponential expression being the only accurate (Figure 3), in the *in vivo* situation the selection of a constitutive equation for the tumor becomes less important.

### Mechanical interactions with the host tissue strongly affect the *in vivo* state of stress and growth rate of the tumor

**Figure 6:**
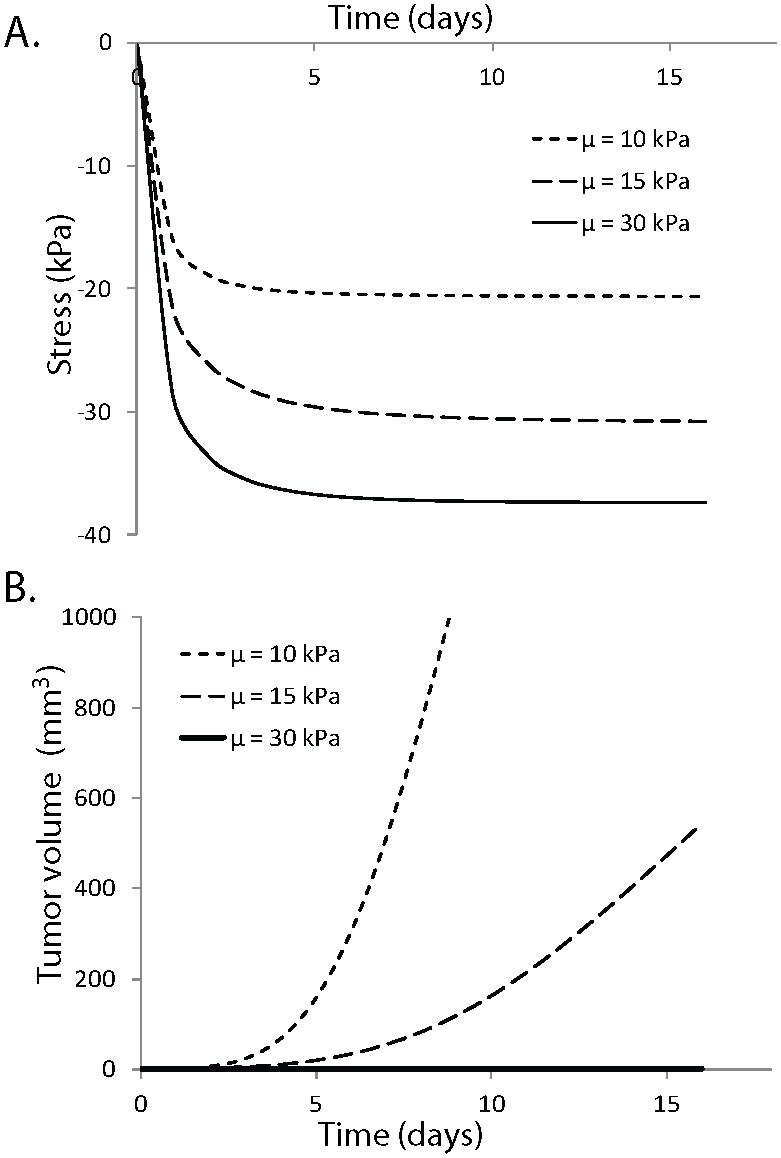
Effect of tumor-host mechanical interactions on tumor state of stress and growth. Dependence of A) state of stress and B) growth rate of tumors on the mechanical properties of the host tissue. The host tissue was modeled as a compressible neo-Hookean material with Poisson’s ratio of 0.2 and three values of the shear modulus were used, *µ* = 10, 15 and 30 kPa. The stiffer the host tissue is, the higher the stress in the tumor and the lower its growth rate becomes.

The generation of solid stress during progression must depend on the resistance from the host tissue. Provided that the tumor is stiffer than the host tissue, as it grows it would displace the surrounding tissue, which in turn would resist to tumor expansion. Therefore, the stress in the tumor should depend on the mechanical properties of the host. In Figure 6A, model predictions for the evolution of the bulk solid stress of the tumor are plotted for three values of the shear modulus of the host tissue, keeping the Poisson’s ratio constant to 0.2. The exponential equation and the parameter values given in Table 2 for the SW620 cell line were used for the tumor. The figure shows that the stiffer the surrounding tissue is, the higher the stress in the tumor becomes, which inhibits tumor growth (Figure 6B).

These results of our mathematical model can explain our previously published *in vivo* data, which show that when the same cancer cell line grows in different host tissues, it exhibits different growth rates [2]. Also, our results are in agreement with *in vitro* studies of cancer cell spheroids grown within an agarose matrix. In these studies, they increased the concentration of agarose to increase the stiffness of the matrix as well as the compressive stress exerted on the cells and found that making the agarose matrix stiffer inhibits or even completely ceases the growth of the spheroids [5]. Finally, our analysis suggests that stiffening of the tumor is essential for the tumor to grow at the expense of the host tissue. Indeed, in many cancers (e.g. various sarcomas, pancreatic, colon and breast cancers) a desmoplastic reaction takes place that results in the production of collagen and other extracellular molecules, which stiffens the tissue. One pathway through which this might occur is the activation of transforming growth factor-β (TGF-β) that regulates the production of matrix-modifying enzymes [11], [39], [40].

**Figure 7:**
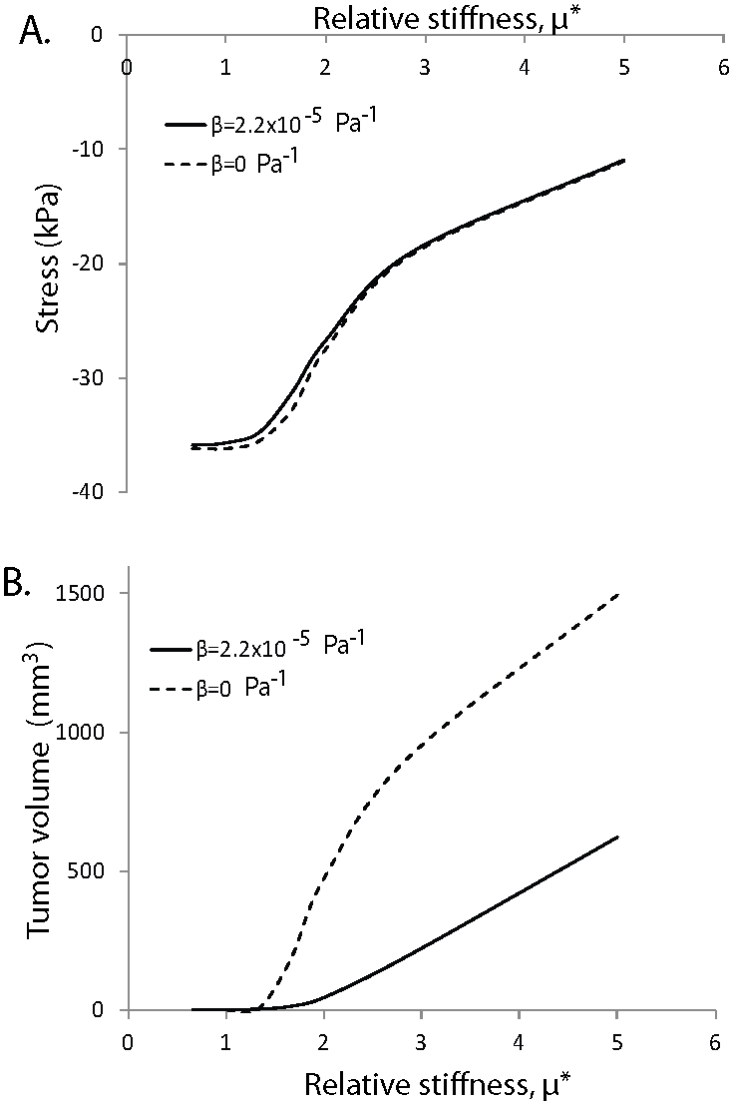
Effect of relative stiffness of the tumor compared to the host tissue on solid stress and tumor growth. Dependence of A) the state of stress and B) tumor volume on the relative stiffness of the tumor compared to the normal tissue, µ*. Relative stiffness is the ratio of the tumor shear modulus to that of the host. Results correspond to day 5 of the simulations. Tumor solid stress increases with stiffening of the surrounding host tissue and reaches a plateau when the stiffness of the tumor becomes the same as or lower than the stiffness of the host (panel A). The tumor has to reach a critical stiffness compared with that of the normal tissue to be able to displace the tissue and grow (panel B).

Finally, we used the mathematical model to investigate how stiff a tumor should become relative to the host tissue, so that it will be able to grow in size. *In vitro* studies using cancer cell spheroids grown in a fibrous matrix [5] have shown the existence of a critical value of the relative stiffness of the tumor compared with the host tissue above which tumors can grow, but quantification of this critical stiffness can be estimated only with mathematical modeling. Figure 7 presents the bulk stress and the volume of the tumor as a function of the relative stiffness, µ*. µ* is defined as the ratio of the tumor’s shear modulus over the shear modulus of the normal tissue. We modeled both tissues with the neo-Hookean equation, the value of the shear modulus given in Table 2 for the SW620 cell line was used for the tumor, while the shear modulus of the normal tissue was varied. The results correspond to day 5 of the simulations and two values of the parameter *β* were used, the one found by fitting the model to the experimental data of Figure 3 and a value of zero, which renders the growth stretch ratio, *λ_g_*, independent from the stress, and thus, the inhibitory effect of stress on tumor growth diminishes. The compressive solid stress in the tumor interior increases as the shear modulus of the host tissue increases (i.e., µ* becomes smaller), but interestingly it reaches a plateau when the shear modulus of the host tissue is equal to or higher than the modulus of the tumor (Figure 7A). Also, in agreement with the *in vitro* studies, Figure 7B suggests that the stiffness of the tumor should exceed a critical value compared with the stiffness of the host tissue, so that the tumor will be able to physically displace the tissue and grow in size. The model further predicts that this critical value of relative stiffness should be on the order of 1.5. It is noted, however, that apart from mechanical factors, biological factors that are not accounted for in our analysis, such as the expression of matrix-modifying agents (e.g., matrix metalloproteinases) or the supply of oxygen and nutrients also affect the progression of the tumor.

## Discussion

Mechanical forces shape the tumor microenvironment and largely determine tumor progression and therapeutic outcome [13]. Despite the important role of the mechanical behavior of solid tumors, to date there is no experimental method to quantify solid stress levels *in vivo*. Only recently, we invented an *ex vivo* technique to quantify the growth-induced, residual component of the solid stress [2]. Therefore, the estimation of solid stress levels during progression requires the use of mathematical modeling. The accuracy of current mathematical models, however, is limited because of a lack of experimental studies to characterize the mechanical response of tumors.

This study had two major objectives. The first objective was to perform unconfined compression experiments in two tumor types in order to measure the visco-elastic response of the tumors, derive their elastic modulus and tan(*δ*) in compression and determine a suitable constitutive equation that reproduces the experimentally measured *ex vivo* elastic response. The second objective was the use of a biomechanical model of tumor growth to determine to what extent the selection of a constitutive equation affects solid stress levels in a growing tumor and what is the contribution of the mechanical interactions with the host tissue to the state of stress and growth of the tumor. We found that solid tumors exhibit a highly nonlinear elastic response in compression, even at very low strains (Figure 2), similar to most soft biological tissues including arteries, tendons and ligaments [36]. The elastic modulus was measured to be 288.3±45.5 kPa for the MCF10CA1a and 186.1±25.9 kPa for the SW620 tumors (Table 1). These values are comparable to the elastic moduli of other soft biological tissues, such as the arterial wall [41], and at least an order of magnitude higher than previous measurements in tumors [26], [27]. The discrepancy with the previous studies is due to the fact that in those measurements a small piece of the tumor (<5 mm) was used as a specimen, while in our study we tested almost the entire tumor. Therefore, our results correspond to the macroscopic, tissue-level, response of the tissue.

We also found that due to the highly nonlinear *ex vivo* elastic response of solid tumors, the commonly used neo-Hookean and Blatz-Ko constitutive equations fail to capture the mechanical behavior of the tumor. An exponential equation more accurately reproduces the experimental response and it should be preferred in mathematical models along with the corresponding mechanical properties, given in Table 2. In the *in vivo* situation, however, the evolution of solid stress in the tumor depends strongly on the properties of the host tissue and the selection of the constitutive equation becomes less important. The fact that variations in the choices of properties and constitutive models all led to the same conclusion is a finding that suggests a fundamental phenomenon and not an artifact of arbitrary modeling choices. It should be noticed, however, that in the current study we used constitutive equations of compressible materials. In previous research [3] we showed that treating tumors as incompressible can lead to higher stresses than the compressive case. Whether solid tumors are compressible or incompressible still remains an open question. Taken together, we conclude that apart from the mechanical behavior of the tumor, one should measure and incorporate into mathematical models the properties and mechanical response of the host tissue as well. Given the direct correlation between mechanical compression and inhibition of cancer cell proliferation [6], the mechanical behavior of the host tissue plays also an important role in the growth rate of the tumor. We highlighted here that the host tissue inhibits tumor growth not only by suppressing cancer cell proliferation but also by resisting to the expansion of the tumor.

The biomechanical model presented in this study is limited in that it does not account for the effect of solid stress on the compression of blood vessels, which reduces perfusion and the supply of oxygen and nutrients. Vascular compression is an indirect way that inhibits tumor growth, independently from the direct effect of solid stress [5], [6], [42]. Incorporation of vessel compression by solid stress would further inhibit the growth rate of the tumor. Also, we did not consider in our analysis the growth-induced, residual component of solid stress. Growth-induced stress contributes less than 30% to the total solid stress of the tumor, which suggests that the external stress by the host tissue is the dominant component of solid stress [3]. Another parameter not considered in our study was the contribution of the interstitial fluid to the total stress and the pathology of cancer [17], [43]. Interstitial fluid pressure is additive to the bulk solid stress to yield the total stress of the tissue. Typical interstitial fluid pressure values in murine tumors are in the range of 1.0–4.0 kPa (10–30 mmHg) [44], much lower than solid stress, and because it is uniformly distributed in the tumor interior has no effect on solid stress levels [3]. Finally, as many other biological tissues, solid tumors are highly heterogeneous structures, consisting of multiple structural components, such as cancer and stromal cells, collagen and hyaluronan [45], [46]. Each of these constituents has a different contribution to the mechanics of a tumor [2] and their concentration and organization in the tissue can vary considerably in the same tumor during progression, between the primary tumor and its metastases and among tumors of different types. Therefore, further studies are required to relate the structure to the mechanical function of tumors. Structure-based models [47], [48] should be better suited to investigate this correlation compared to continuum-level constitutive equations, like these employed in our study.

## Conclusions

The mechanical behavior of solid tumors is highly nonlinear even at low strains and it can be better described by an exponential constitutive equation. The state of stress of a growing tumor, however, does not depend so much on the selection of the constitutive equation, but it is determined by the mechanical interactions with the surrounding host tissue, and particularly by the mechanical properties of the host. According to our calculations and in agreement with *in vitro* studies, solid tumors must exceed a critical stiffness compared with the host tissue in order to be able to displace the host tissue and grow. With the use of our mathematical model, we estimated this critical value to be on the order of 1.5. Therefore, the direct effect of solid stress on tumor growth involves not only the inhibitory effect of stress on cancer cell proliferation and the induction of apoptosis [6], but also the resistance of the surrounding tissue to tumor expansion. Conclusively, our model and findings provide important insights about the role of host tissue-tumor interactions in cancer progression and may serve as the basis for the design of novel therapeutic strategies.

## Supporting Information

File S1 **Description of the mathematical model, Figure S1 and Figure S2.**
(DOCX)
