# Supplementary material for: Role of Constitutive Behavior and Tumor-Host Mechanical Interactions in the State of Stress and Growth of Solid Tumors
Source: PLoS One. 2014 Aug 11;9(8):e104717. doi: 10.1371/journal.pone.0104717 (PMC4128744; doi:10.1371/journal.pone.0104717)
Supplement: File S1 — Description of the mathematical model, Figure S1 and Figure S2. (DOCX) [file pone.0104717.s001.docx]

**Supporting Information**

**Role of constitutive behavior and tumor-host mechanical interactions in the state of stress and growth of solid tumors**

C. Voutouri, F. Mpekris, P. Papageorgis, A. D. Odysseos, and T. Stylianopoulos

**Description of the mathematical model**

The model is based on the multiplicative decomposition of the deformation gradient tensor:

$\mathbf{F}=\mathbf{F}_{e}\mathbf{F}_{g}\mathbf{F}_{r}$, (1)

where **F**_e_ is the elastic component of the deformation gradient tensor that accounts for interactions with the normal tissue and ensures the continuity of the body, **F**_r_ is the component that accounts for the generation of growth-induced/residual stresses and **F**_g_ is the component that accounts for cancer cell proliferation.

We took **F**_r_=**I** because no measurable growth-induced stress was observed in the experiments [[1](#_ENREF_1),[2](#_ENREF_2)]. Tumor growth was assumed to be isotropic and governed by Gompertz law:

$\mathbf{F}_{g}=\lambda_{g}I$ and $\frac{d\lambda_{g}}{dt}=-a\lambda_{g}\log\left(\lambda_{g}/\lambda_{\max}\right)$, (2)

where λ_g_, is the growth "stretch ratio".

Furthermore, *a* describes the growth rate of the cells and λ_max_ is a maximum growth stretch ratio. We further assumed that the parameter *a* depends on the solid stress levels through a linear relationship [[3](#_ENREF_3)], i.e.,

$a=a_{o}\left(1+\beta\bar{\sigma}\right)$ (4)

where *a*_o_ is the proliferation rate at zero stress, *β* is a constant that describes the dependence of *a* on stress and , is the average bulk stress. Only compressive stresses were assumed to inhibit tumor growth (15, 16) and thus, $a=a_{o}$when the bulk stress is tensile.

Therefore, the elastic component of the deformation gradient tensor was determined by

$\mathbf{F}_{e}=\mathbf{FF}_{g}^{-1}$ (5)

and by definition$F_{ij}=\frac{\partial x_{i}}{\partial X_{j}}$. COMSOL calculates the overall deformation gradient tensor **F** and thus, we have to employ Eq. 5 in order to calculate the elastic component of **F**, which is being used for the calculation of the stress tensor [[4](#_ENREF_4)]: $\mathbf{\sigma}=J_{e}^{-1}\mathbf{F}_{e}\frac{\partial W}{\partial\mathbf{F}_{e}^{T}}$

where W is the strain energy density function and J_e_ the determinant of **F**_e_. The strain energy density functions employed in the current study were:

neo-Hookean: $W=0.5\mu\left(-3+II_{1}\right)+0.5\kappa\left(-1+J_{e}\right)^{2}$ (6)

Blatz-Ko: $W=\frac{G_{t}f}{2}\left(I_{1}-3-\frac{2}{q}\left(I_{3}^{q/2}-1\right)\right)+\frac{G_{t}(1-f)}{2}\left(\frac{I_{2}}{I_{3}}-3-\frac{2}{q}\left(I_{3}^{-q/2}-1\right)\right)$ (7)

exponential: $W=\text{A}_{1}\text{(e}^{\text{C}_{1}\text{(-3+II}_{1}\text{)}}\text{ -1) + A}_{2}\text{(-1+J}_{e}\text{)}^{2}$ (8)

where the mechanical properties of the neo-Hookean model are the shear modulus μ and bulk modulus κ. The properties of the Blatz-Ko model are the shear modulus G_t_, $f\in[0 1]$ and q<0 are dimensionless parameters. For isotropic materials$f=1$, and thus only the first term of the right hand side of Eq. 6 was considered in our study. The parameters of the exponential equation are the constants A_1_, A_2_ and C_1_. $II_{1}=I_{1}J_{e}^{-2/3}$. *I_1_*, *I_2_* and *I_3_* are the first invariant of the right Cauchy-Green deformation tensor, which is evaluated from the elastic part of the deformation gradient tensor, **F**_e_.

Finally the linear momentum balance for a quasi-static problem is

$\nabla\cdot\mathbf{\sigma}=0$ (9)

**COMSOL implementation of the model**

The solution of the linear momentum balance in COMSOL v. 3.5 is based on the 1st Piola-Kirchhoff stress tensor, which is calculated as [[4](#_ENREF_4)]:

$\mathbf{P}=JJ_{e}^{-1}\frac{\partial W}{\partial\mathbf{F}}$ (10)

where *J* is the determinant of the deformation gradient tensor, J_e_ the determinant of the elastic component **F**_e_ and W the strain energy density function.

However, COMSOL by default calculates a pseudo 1st Piola-Kirchhoff stress tensor:

$\overline{\mathbf{P}}=\frac{\partial W}{\partial\mathbf{F}}$ (11)

and thus, the user has to intervene to the model equations in order to enforce the use of Eq. 10.

The model consisted of two domains: the tumor and the surrounding normal tissue. Because of symmetry only one eighth of the domain was considered (Supplementary Fig. S2). The "Structural Mechanics" module was employed to solve the linear momentum balance in both domains. The "General Form PDE" module was employed to solve for the growth stretch ratio (Equation 2) in domain of the tumor. As for initial conditions, the growth stretch ratio was taken to be unity and the momentum balance along with Eq. 2 were solved simultaneously. The boundary conditions applied to the problem are shown in Supplementary Fig. S2. Symmetry ("Roller") and stress-free ("Free") conditions were used. At the interface between the tumor and normal tissue the continuity of the stress and displacement were applied by the software automatically. Large deformations were enabled, the solid mechanics problem was solved on the reference frame (Lagrangian description), while the differential equation for the growth stretch ratio was solved on the deformed frame.

**References**

**1. Stylianopoulos T, Martin JD, Chauhan VP, Jain SR, Diop-Frimpong B, et al. (2012) Causes, consequences, and remedies for growth-induced solid stress in murine and human tumors. Proceedings of the National Academy of Sciences of the United States of America 109: 15101-15108.**

**2. Stylianopoulos T, Martin JD, Snuderl M, Mpekris F, Jain SR, et al. (2013) Coevolution of solid stress and interstitial fluid pressure in tumors during progression: Implications for vascular collapse. Cancer research 73: 3833-3841.**

**3. Roose T, Netti PA, Munn LL, Boucher Y, Jain RK (2003) Solid stress generated by spheroid growth estimated using a linear poroelasticity model. Microvasc Res 66: 204-212.**

**4. Taber LA (2008) Theoretical study of Beloussov's hyper-restoration hypothesis for mechanical regulation of morphogenesis. Biomech Model Mechanobiol 7: 427-441.**

**Supporting Figures**


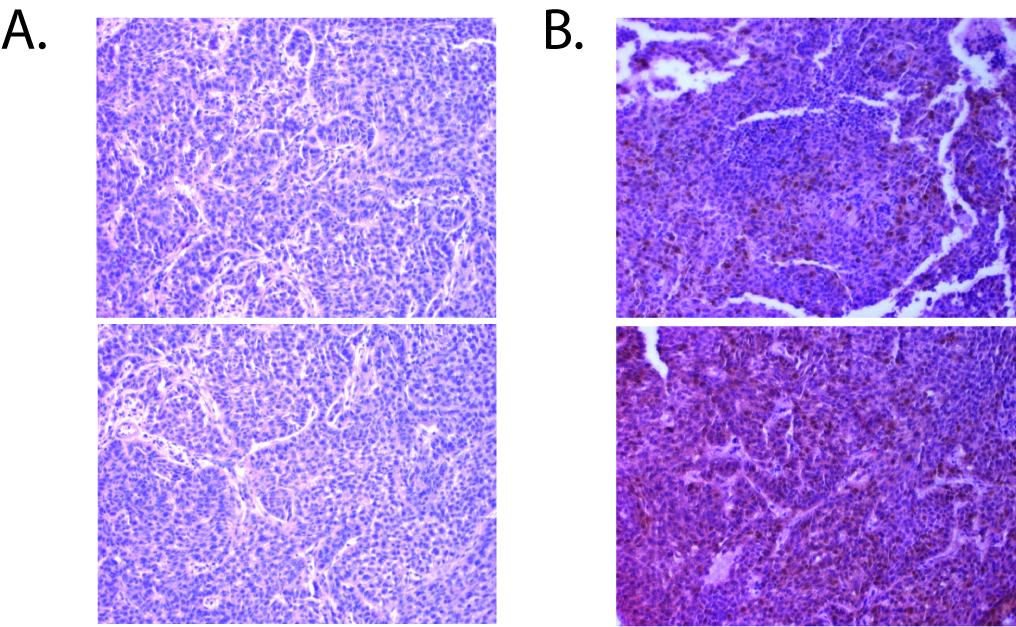


**Figure S1**. Representative Hematoxylin and Eosin (20X) images of MCF10CA1a (A) and SW620 (B) tumor sections showing the malignant state of the cells.


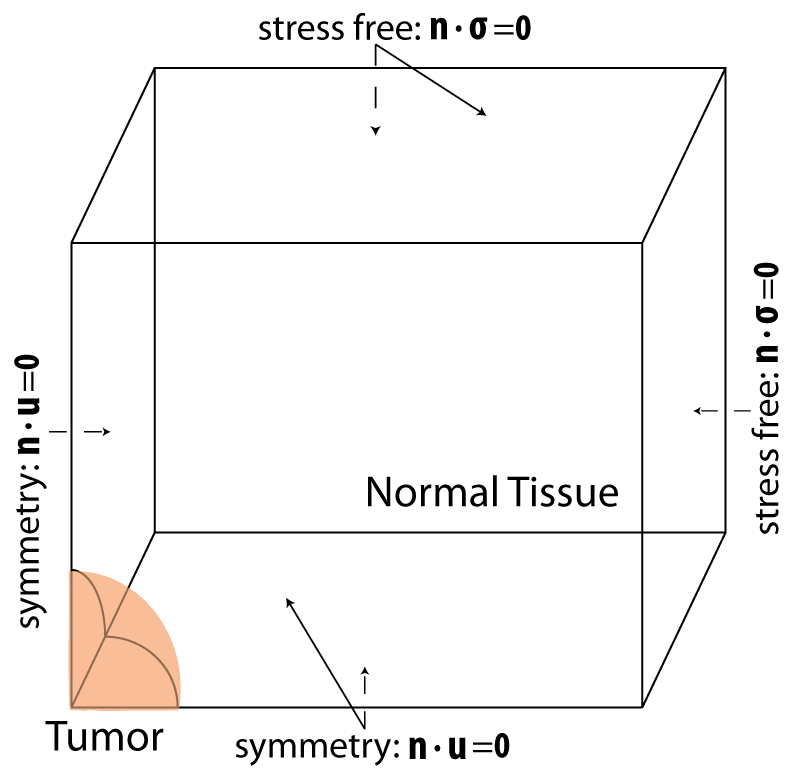


**Figure S2**. Boundary conditions employed. Due to symmetry the one eight of the domain was solved. **n** is the unit normal vector and **u** is the displacement vector. The continuity of the displacements and the normal stress at the tumor-host interface are implemented automatically by the software.
